# The growing controversy about growth charts: WHO or regional?

**DOI:** 10.1186/1687-9856-2013-S1-O6

**Published:** 2013-10-03

**Authors:** Vaman Khadilkar

**Affiliations:** 1 Pediatric Endocrinologist, HCJMRI and Bharati VIdyapeeth Medical College, Pune, India

## Introduction

Since production of WHO Multicenter Growth Reference Study (MGRS) growth standards in 2006, many countries have adopted the WHO charts for under five children [1]. In UK, WHO growth charts are used until 4 years [2] and in US only up to 2 years of age. Over-diagnosis of stunting and underweight in Asian children is likely with the use of these standards as Asian children are still thinner and lighter.

## Growth standard vs. reference

A growth reference simply describes the growth of a sample of individuals, whereas a standard describes the growth of a ‘healthy’ population and suggests an aspirational model. WHO growth charts are growth standards. A reference is representative of the existing growth pattern of children and allows us to study the secular trends in height, weight and obesity.

## Advantages of WHO growth charts

WHO growth standards have given a platform to compare growth of under five children across all races and ethnicity against a single standard, thus assessment becomes objective and easy. They show more physiological growth pattern as the children in MGRS study were breast fed and hence leaner, promoting prevention of obesity from a younger age. The MGRS provides an unsurpassed foundation for a growth standard based on healthy children living under conditions that favored the achievement of full genetic potential.

## Disadvantages of WHO growth standards

In developing nations the WHO 2006 standards tend to over-diagnose stunting and wasting. In a nationwide study done by the author on apparently healthy affluent Indian children the percentage of stunting was 13.6% for boys and 11.2% for girls and that for wasting was 8.5% for boys vs. 10.4% for girls [3]. Similar concerns are expressed by authors from other developing countries such as Indonesia [4], and Malawi [5]. In a study done by Kerac M et al on data from 21 countries it was concluded that use of WHO standards to define wasting results in a greater disease burden, in children under the age of 6 months[6] .

## Conclusion

WHO 2006 growth standards are useful for comparison of growth of children around the world but caution regarding referral for investigations of failure to thrive, changing infant feeding policies and intervention programs based on WHO 2006 standards for the developing part of the world is needed at least for the present time.
